# A novel *PRRX1* loss-of-function variation contributing to familial atrial fibrillation and congenital patent ductus arteriosus

**DOI:** 10.1590/1678-4685-GMB-2021-0378

**Published:** 2022-03-30

**Authors:** Zun-Ping Ke, Gao-Feng Zhang, Yu-Han Guo, Yu-Min Sun, Jun Wang, Ning Li, Xing-Biao Qiu, Ying-Jia Xu, Yi-Qing Yang

**Affiliations:** 1 Fudan University, Shanghai Fifth People’s Hospital, Department of Geriatrics, Shanghai, China.; 2 Fudan University, Shanghai Fifth People’s Hospital, Department of Cardiology, Shanghai, China.; 3 Fudan University, Shanghai Fifth People’s Hospital, Center for Complex Cardiac Arrhythmias of Minhang District, Shanghai, China.; 4 Fudan University, Shanghai Jing'an District Central Hospital, Department of Cardiology, Shanghai, China.; 5 Shanghai Jiao Tong University, Shanghai Chest Hospital, Department of Cardiology, Shanghai, China.; 6 Fudan University, Shanghai Fifth People’s Hospital, Cardiovascular Research Laboratory, Shanghai, China.; 7 Fudan University, Shanghai Fifth People’s Hospital, Central Laboratory, Shanghai, China.

**Keywords:** Cardiac arrhythmia, congenital heart defect, medical genetics, PRRX1, reporter gene analysis

## Abstract

Atrial fibrillation (AF) represents the most common type of sustained cardiac arrhythmia in humans and confers a significantly increased risk for thromboembolic stroke, congestive heart failure and premature death. Aggregating evidence emphasizes the predominant genetic defects underpinning AF and an increasing number of deleterious variations in more than 50 genes have been involved in the pathogenesis of AF. Nevertheless, the genetic basis underlying AF remains incompletely understood. In the current research, by whole-exome sequencing and Sanger sequencing analysis in a family with autosomal-dominant AF and congenital patent ductus arteriosus (PDA), a novel heterozygous variation in the *PRRX1* gene encoding a homeobox transcription factor critical for cardiovascular development, NM_022716.4:c.373G>T;p.(Glu125^*^), was identified to be in co-segregation with AF and PDA in the whole family. The truncating variation was not detected in 306 unrelated healthy individuals employed as controls. Quantitative biological measurements with a reporter gene analysis system revealed that the Glu125^*^-mutant *PRRX1* protein failed to transactivate its downstream target genes *SHOX2* and *ISL1*, two genes that have been causally linked to AF. Conclusively, the present study firstly links *PRRX1* loss-of-function variation to AF and PDA, suggesting that AF and PDA share a common abnormal developmental basis in a proportion of cases.

## Introduction

Atrial fibrillation (AF), characteristic of rapid and disorganized electrical activation and inefficient contraction of the atria, is the most common form of clinical dysrhythmia that affects approximately 1% of the general population globally (Tian *et al.*, 2020; Zhang *et al.*, 2021). Its global prevalence is low in individuals aged <40 years, but increases abruptly beyond the age of 65 years, reaching over 10% in subjects ≥80 years old (Huang *et al.*, 2020). The lifetime for development of AF is ~25% in subjects ≥40 years of age and ~37% in those ≥55 years of age (Lloyd-Jones *et al.*, 2014; Weng *et al.*, 2018). Given that about one third of the total AF population is silent or subclinical asymptomatic, the global prevalence of AF is certainly underestimated (Dilaveris and Kennedy, 2017). AF confers a significantly increased risk for ischemic or hemorrhagic stroke, dementia, venous thromboembolism, acute myocardial infarction, congestive heart failure and premature death with substantial socioeconomic costs (Kornej *et al.*, 2020). Nevertheless, existing therapeutic regimens for AF are considerably limited in effectiveness and seldom curative, which reflects a poor understanding of the molecular mechanisms underpinning this complex supraventricular arrhythmia (Huang *et al.*, 2020).

Epidemiological investigations have revealed that environmental risk factors predispose to the occurrence and perpetuation of AF, such as advancing age, obesity, obstructive sleep apnea, diabetes mellitus, arterial hypertension, valvular heart diseases, coronary artery disease, dilated cardiomyopathy, hyperthyroidism, heart failure, smoking, alcohol consumption, psychological stress and extreme sports (January *et al.*, 2014; Kornej *et al.*, 2020). However, in ~30% of patients, no well-established cardiovascular pathologies or precipitating factors for AF can be identified, which suggests possible genetic basis underlying AF (Ragab *et al.*, 2020). During the past two decades, multiple epidemiological investigations have demonstrated familial aggregation of individuals with AF and the heritability of AF has been estimated to be as high as 62%, highlighting a strong heritable component responsible for AF (Roselli *et al.*, 2020). By genotyping with a few hundred polymorphic microsatellite markers scattered throughout the genome and genetic linkage analysis of AF families, Brugada and his partners located the first locus for AF at human chromosome 10q22-q24 (Brugada *et al.*, 1997). Subsequently, similar genetic studies linked more genetic loci to AF, including human chromosome 5p13, 5p15, 6q14-16, 10p11-q21 and 20q12-13 (Ellinor *et al.*, 2003; Oberti *et al.*, 2004; Volders *et al.*, 2007; Darbar *et al.*, 2008). By genetical analysis of a large Chinese family inflicted with AF, Chen and his coworkers mapped a new locus for AF to chromosome 11p15.5 and in this chromosomal region discovered the first AF-causative gene, S140G-mutant *KCNQ1*, which encodes an α subunit of voltage-gated potassium channel (Chen *et al.*, 2003). Functional analysis of the S140G-mutant *KCNQ1* unveiled a gain-of-function impact on the currents of *KCNQ1*/KCNE1 and *KCNQ1*/KCNE2 channels, which significantly shorten the action potential duration of atrial myocytes thereby increasing the vulnerability to AF (Chen *et al.*, 2003). Up to now, in addition to the association of ~140 genetic loci with increased predisposition to AF revealed by genome-wide association studies (Kim *et al.*, 2021), rare variations in over 50 distinct genes have been discovered to contribute to AF, amidst which the majority encode cardiac potassium ion channels, sodium channels, gap junction channels, calcium channels, signaling molecules, structural proteins and transcription factors (Choi *et al.*, 2020; Ghazizadeh *et al.*, 2020; Hansen *et al.*, 2020; Huang *et al.*, 2020; Jiang *et al.*, 2020; Ragab *et al.*, 2020; Roselli *et al.*, 2020; van Ouwerkerk *et al.*, 2020; Wu *et al.*, 2020; Yang *et al.*, 2020; Chalazan *et al.*, 2021; Lazarte *et al.*, 2021; Li *et al.*, 2021a, b; Ziki *et al.*, 2021). Interestingly, multiple variations in or near the *PRRX1* gene, has recently been associated with an enhanced susceptibility to AF in humans (Tucker *et al.*, 2017; Guo *et al.*, 2021; Wu *et al.*, 2021). However, due to pronounced genetical heterogeneity, the genetic determinants underlying AF remain largely elusive. This study was sought to identify a novel genetic variation predisposing to AF.

## Material and Methods

### Recruitment and clinical evaluation of study participants

For this investigation, a three-generation family affected with AF and congenital patent ductus arteriosus (PDA) was identified, from which 18 available family members were enlisted. A total of 306 unrelated healthy volunteers, who had neither AF nor congenital heart defect (CHD), the most common type of birth defects (Oliveira-Brancati *et al.*, 2020; Virani *et al.*, 2021), were enrolled as control subjects. All study participants experienced a comprehensive clinical assessment, including review of medical histories, physical examination, electrocardiography and echocardiography as well as routine laboratory tests. The healthy control individuals were exactly matched with the cases for gender, ethnicity and age. Clinical diagnosis and classification of AF or CHD were made as previously described (January *et al.*, 2014; Benjamin *et al.*, 2019; Wang *et al.*, 2020; Zhao *et al.*, 2021). This case-control research was carried out in conformity with the ethical tenets outlined in the Declaration of Helsinki and was approved by the Medical Ethics Committee of Shanghai Chest Hospital (with an approval number of KS1101). Prior to collection of peripheral venous blood samples, informed consent was provided by the study participants or their parents.

### Whole-exome sequencing and bioinformatical analysis

**Figure 1 -:**
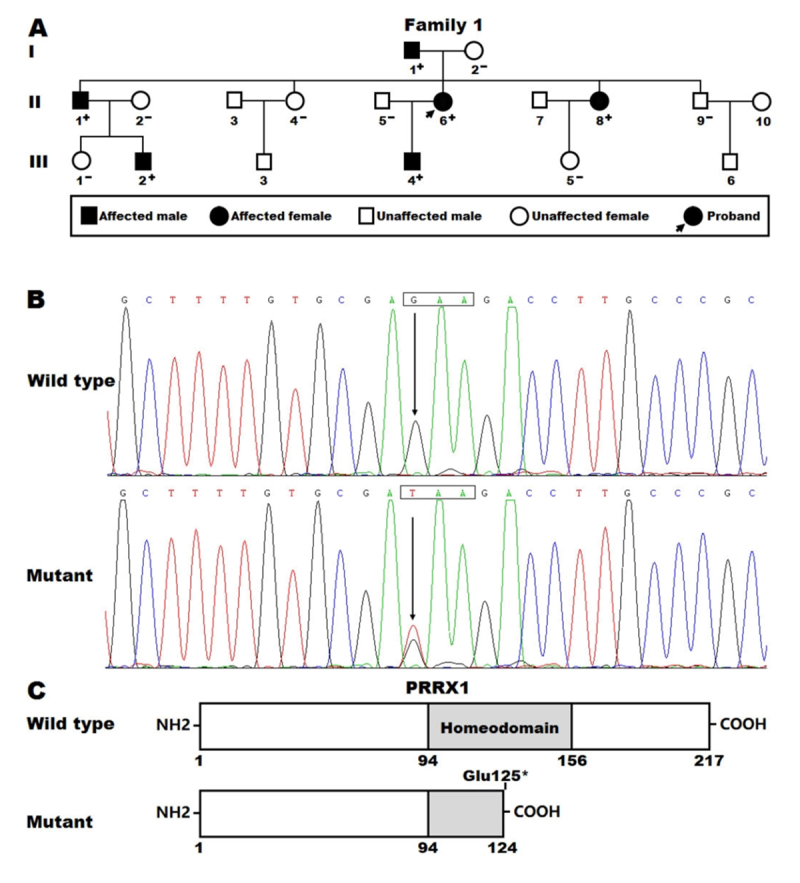
A new *PRRX1* variation predisposing to familial atrial fibrillation and congenital heart defect. (A) Pedigree structure of the family inflicted with atrial fibrillation and congenital heart disease. “+”, carriers of the *PRRX1* variation; “-”, non-carriers. (B) Sequence electropherogram traces showing the heterozygous *PRRX1* variation (mutant) as well as its homozygous wild-type control base (wild type). A rectangle delimits a codon comprising three nucleotides. (C) Schemas exhibiting the structural domains of the *PRRX1* proteins. NH2, amino-terminus; COOH, carboxyl-terminus.

Genomic DNA was extracted from the venous blood leucocytes of every test person utilizing a genomic DNA extraction kit (Promega, USA). Whole-exome sequencing (WES) and bioinformatical analysis in five affected family members (I-1, II-1, II-6, III-2 and III-4, Figure 1A) and four unaffected family members (I-2, II-2, II-5 and III-1, Figure 1A) were performed as described previously (Di *et al.*, 2020; Qiao *et al.*, 2020; Linhares *et al.*, 2021; Wang *et al.*, 2021; Xian *et al.*, 2021). In brief, each exome library was constructed using 5 μg of genomic DNA from a study subject, enriched by ligation-mediated polymerase chain reaction (PCR) and captured with the SureSelect Human All Exon V6 Kit (Agilent Technologies, USA). Each exome library was sequenced on the Illumina HiSeq 2000 Genome Analyzer (Illumina, USA) by utilizing the HiSeq Sequencing Kit (Illumina, USA) as per the manufacturer’s instructions. Raw image files were processed using the software Pipeline (Illumina, USA) to call bases and the sequences of each subject were generated as a set of reads. By using the Burrows-Wheeler Aligner (BWA) software (Li and Durbin, 2009, 2010), sequencing reads were aligned to the sequences of referential human genome (GRCh37/hg19). Variation calling was performed with the SAMtools (Sequence Alignment/Map Tools, version 0.1.18) software (Li *et al.*, 2009; Li, 2011) and the Genome Analysis Toolkit (GATK, version 4.0.10.1) software (McKenna *et al.*, 2010). The genetic variants that passed the pedigree analysis with any reasonable inheritance pattern of AF and PDA were annotated with the ANNOVAR (annotation of variance, version 20170221) software (Wang *et al.*, 2010). Deleterious variations with a minor allele frequency of <0.001 (in such databases as the Genome Aggregation Database and the Single Nucleotide Polymorphism database) annotated by ANNOVAR were selected as candidate disease-causing variants subject to confirmation by Sanger sequencing analysis in the whole family. The entire coding region and splicing donors/acceptors of the gene harboring a confirmed candidate causative variant were PCR-sequenced in all the available family members and 306 unrelated healthy persons. For an identified rare damaging variation, the Single Nucleotide Polymorphism database (https://www.ncbi.nlm.nih.gov/), the 1000 Genomes Project database (https://www.internationalgenome.org), the Human Gene Mutation Database (http://www.hgmd.cf.ac.uk/ac/index.php), and the UK Biobank database (https://www.ukbiobank.ac.uk/) were consulted to check whether it was novel.

### Construction of recombinant expression plasmids

The recombinant eukaryotic expression plasmid *PRRX1*-pcDNA3.1 expressing human wild-type *PRRX1* was constructed as previously described (Guo *et al.*, 2021). The Glu125^*^-mutant *PRRX1*-pcDNA3.1 was generated by site-targeted mutagenesis utilizing a complimentary pair of primers (forward primer: 5'-GATGCTTTTGTGCGATAAGACCTTGCCCGCC-3'; reverse primer: 5'-GGCGGGCAAGGTCTTATCGCACAAAAGCATC-3') and a site-directed mutagenesis kit (Stratagene, USA) following the manufacturer’s instructions. The Glu125^*^-mutant *PRRX1*-pcDNA3.1 underwent selection by *DpnI* (NEB, Hitchin, UK) and was confirmed by sequencing analysis. The *SHOX2*-luciferase (*SHOX2*-luc) and *ISL1*-luciferase (*ISL1*-luc) reporter plasmids, which both express firefly luciferase, were created as described elsewhere (Guo *et al.*, 2021).

### Cellular transfection with expression plasmids and dual-luciferase assay

Hela cells were cultivated in Dulbecco’s modified Eagle’s medium (Invitrogen, USA) containing 10% fetal calf serum (Thermo Fisher Scientific, USA) together with 1% penicillin/streptomycin (Thermo Fisher Scientific, USA) in an incubator with an air of 5% CO₂ at 37 °C. Hela cells were grown in a 12-well plate at an initial density of 1×10^5^ cells per well 24 h before transient transfection. Cells were transfected with various amounts of expression plasmids as described previously (Guo *et al.*, 2021). The plasmid pGL4.75 (Promega, USA) expressing renilla luciferase was co-transfected as an internal control to normalize transfection efficiency. The activities of firefly and renilla luciferases were measured on a luminometer (Promega, USA) employing a dual-luciferase reporter assay kit (Promega, USA). The activity of a promoter was expressed as fold activation of firefly luciferase relative to renilla luciferase. For each plasmid, three independent transfections were performed and the resultant data for promoter activity were presented as mean ± standard deviation (SD) of three independent transfection experiments.

### Statistical analysis

Unpaired Student’s *t*-test was applied to the comparison of promoter activities between two groups. A two-tailed *p*<0.05 was considered to indicate statistical difference.

## Results

### Clinical characteristics of the pedigree with AF and PDA

As shown in in Figure 1A, a three-generation pedigree with high incidence of AF and PDA was recruited, which comprised 18 living family members, of whom 6 members, including 4 male members and 2 female members with a mean age of 42 years ranging from 19 to 71 years, were diagnosed with AF and PDA in terms of the electrocardiographic and echocardiographic findings. Within this family, AF and PDA were inherited in an autosomal-dominant mode with complete penetrance. Of note, in this pedigree AF began with paroxysmal episodes, but in two family members (members II-1 and II-6) AF became persistent and in one family member (member I-1) AF became permanent over time. No family members had well-established environmental risk factors prone to AF, such as arterial hypertension, valvular heart disease, coronary heart disease, pulmonary heart disease, hyperthyroidism, diabetes mellitus nor obstructive sleep apnea. The proband (member II-6), a forty-three-year-old female member with nineteen years of AF history, was hospitalized due to recurrent syncope and received a successful radiofrequency ablation of AF. The proband’s other affected relatives had a history of taking anti-arrhythmic drugs but none of them underwent interventional treatment for AF at the time of enrollment. Additionally, catheter-based closure of PDA was performed in all the affected family members before 6 years of ages except for family member I-1, who underwent closure of PDA at the age of 22. The unaffected family members (six male members and six female members with an average age of 40 years varying from 15 to 68 years) had neither a history of AF nor a history of CHD, with their electrocardiograms and echocardiograms being normal. The clinical features of the pedigree members suffering from AF and PDA are provided in Table 1.

**Table 1 -:** Clinical features of the family members with atrial fibrillation and patent ductus arteriosus caused by the *PRRX1* variation, NM_022716.4:c.373G>T;p.(Glu125^*^).

Subject information			Phenotype		Electrocardiogram			Echocardiogram	
Identity (Family 1)	Gender	Age (years)	AF (clinical classification)	CHD (anatomic type)	Heart rate (beats/min)	QRS interval (ms)	QTc (ms)	LAD (mm)	LVEF (%)
I-1	M	71	Permanent	PDA	81	113	464	45	58
II-1	M	48	Persistent	PDA	91	84	432	38	60
II-6	F	43	Persistent	PDA	93	90	445	36	64
II-8	F	40	Paroxysmal	PDA	79	109	416	37	66
III-2	M	21	Paroxysmal	PDA	118	84	468	34	68
III-4	M	19	Paroxysmal	PDA	88	78	427	32	65

AF, atrial fibrillation; CHD, congenital heart disease; F, female; LAD, left atrial diameter; LVEF, left ventricular ejection fraction; M, male; PDA, patent ductus arteriosus; QTc, corrected QT interval.

### Discovery of a new causative variation in *PRRX1*

WES was conducted in five affected family members (I-1, II-1, II-6, III-2 and III-4, Figure 1A) and four unaffected family members (I-2, II-2, II-5 and III-1, Figure 1A), generating a mean of 22.9 Gb of sequence for each family member, with ~97% mapping to the referential human genome (GRCh37/hg19) as well as ~73% mapping to the target DNA sequences. An average of 18,632 exonic variations (range 17,105-19,148) per family member passed filtering by inheritance model, of which 12 heterozygous nonsense and missense variations passed filtering by ANNOVAR, shared by the five affected family members and predicted to be pathogenic variants, with minor allele frequencies of <0.001 (as summarized in Table 2). Sanger sequencing analysis in the family revealed that only the variant chr1:170,688,998G>T (GRCh37/hg19: NC_000001.10), equivalent to chr1:170,719,857G>T (GRCh38/hg38: NC_000001.11) or NM_022716.4:c.373G>T;p.(Glu125^*^) in the *PRRX1* gene, was in co-segregation with AF and PDA in the whole family, with complete penetrance. The electropherogram traces exhibiting the heterozygous *PRRX1* variation as well as its homozygous wild-type sequence (used as a control) are exhibited in Figure 1B. The schemas displaying the homeobox domains of wild-type and mutant *PRRX1* proteins are shown in Figure 1C. The truncating variation was neither observed in 306 unrelated healthy individuals, nor found in the Single Nucleotide Polymorphism database, the Single Nucleotide Polymorphism database, the Human Gene Mutation Database, the 1000 Genomes Project database, and the UK Biobank database, indicating it was a novel variation.

**Table 2 -:** Nonsynonymous variations in the candidate genes for familial atrial fibrillation and congenital patent ductus arteriosus identified by whole-exome sequencing analysis.

Chr	Position (GRCh37/hg19)	Ref	Alt	Gene	Variant
1	45,101,789	C	G	RNF220	NM_018150.4: c.1081C>G; p.(Gln361Glu)
1	170,688,998	G	T	*PRRX1*	NM_022716.4: c.373G>T; (p.Glu125^*^)
1	219,383,960	T	A	LYPLAL1	NM_138794.5: c.448T>A; p.(Phe150Ile)
2	125,204,482	G	T	CNTNAP5	NM_130773.4: c.886G>T; p.(Gly296Cys)
3	44,612,251	C	T	ZKSCAN7	NM_018651.4: c.1649C>T; p.(Pro550Leu)
4	140,811,628	G	T	MAML3	NM_018717.5: c.962C>A; p.(Pro321His)
6	155,574,141	G	A	TIAM2	NM_012454.4: c.4179G>A; p.(Trp1393^*^)
10	13,838,538	A	G	FRMD4A	NM_018027.5: c.257T>C; p.(Phe86Ser)
12	66,742,977	T	G	GRIP1	NM_021150.4: c.3053T>G; p.(Leu1018Arg)
15	29,385,289	T	C	APBA2	NM_005503.3: c.1081T>C; p.(Cys361Arg)
17	4,100,789	C	A	ANKFY1	NM_016376.5: c.982C>A; p.(His328Asn)
19	54,080,349	A	T	ZNF331	NM_018555.6: c.535A>T; p.(Lys179^*^)

Chr, chromosome; Ref, reference; Alt, alteration.

### No transcriptional activation of *SHOX2* by the Glu125^*^-mutant *PRRX1* protein

**Figure 2 -:**
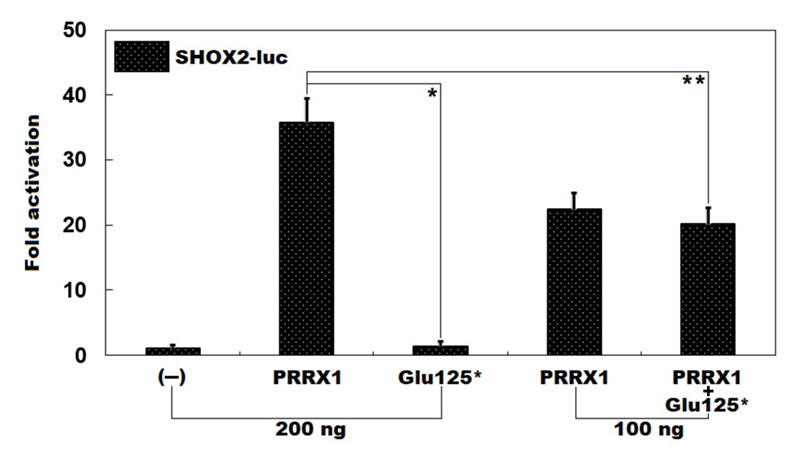
Failure to transactivate *SHOX2* by Glu125^*^-mutant *PRRX1*. Dual-luciferase reporter assays unveiled that in cultured Hela cells overexpressing various recombinant expression plasmids, Glu125^*^-mutant *PRRX1* (Glu125^*^) failed to transactivate the promoter of the *SHOX2* gene, singly or together with wild-type *PRRX1* (*PRRX1*). The symbols ^*^ and ^**^ mean p<0.001 and p<0.005, respectively, in comparison with wild-type *PRRX1* (200 ng).

As shown in Figure 2, in cultured Hela cells overexpressing various recombinant expression plasmids, 200 ng of wild-type *PRRX1* plasmid and the same amount (200 ng) of Glu125^*^-mutant *PRRX1* plasmid transactivated the promoter of *SHOX2* by 36 folds and 1 fold, respectively (wild-type *PRRX1* vs. Glu125^*^-mutant *PRRX1*: *t* = 15.7809, *p* = 0.00009). When 100 ng of wild-type *PRRX1* plasmid and the same amount (100 ng) of Glu125^*^-mutant *PRRX1* plasmid were used together, the induced transcriptional activity was 20-fold (wild-type *PRRX1* plasmid plus empty plasmid vs. wild-type *PRRX1* plasmid plus Glu125^*^-mutant *PRRX1* plasmid: *t* = 6.01821, *p* = 0.00384).

### No transcriptional activation of *ISL1* by the Glu125^*^-mutant *PRRX1* protein

**Figure 3 -:**
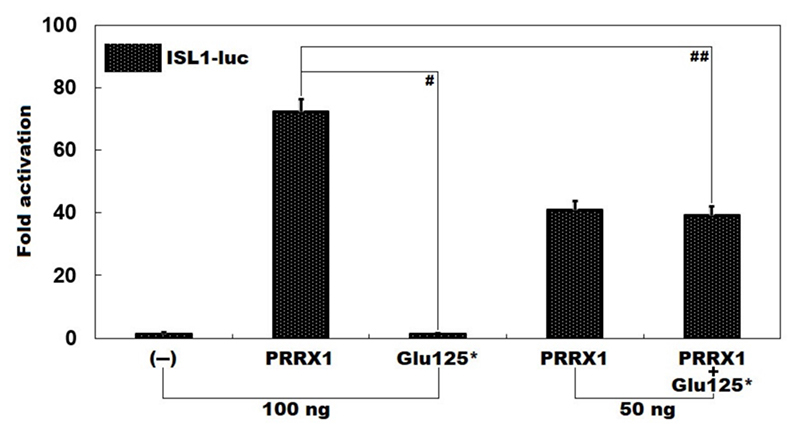
No transcriptional activation on the promoter of *ISL1* by Glu125^*^-mutant *PRRX1*. Biological measurement of the transactivation of the *ISL1* promoter-driven luciferase in cultivated Hela cells expressing various expression plasmids by wild-type *PRRX1* (*PRRX1*) or Glu125^*^-mutant *PRRX1* (Glu125^*^), alone or in combination, revealed that Glu125^*^ lost the ability to transcriptionally activate the promoter of the *ISL1* gene. Here # and ## mean p<0.0001 and p<0.0005, respectively, in comparison with wild-type counterpart (100 ng).

As shown in Figure 3, in cultured Hela cells overexpressing various recombinant expression plasmids, 100 ng of wild-type *PRRX1* plasmid and the same amount (100 ng) of Glu125^*^-mutant *PRRX1* plasmid transactivated the promoter of *ISL1* by 72 folds and 1 fold, respectively (wild-type *PRRX1* vs. Glu125^*^-mutant *PRRX1*: *t* = 29.5856, *p* = 0.00001). When 50 ng of wild-type *PRRX1* plasmid and the same amount (50 ng) of Glu125^*^-mutant *PRRX1* plasmid were used in combination, the induced transcriptional activity was 39-fold (wild-type *PRRX1* plasmid plus empty plasmid vs. wild-type *PRRX1* plasmid plus Glu125^*^-mutant *PRRX1* plasmid: *t* = 11.3859, *p* = 0.00034).

## Discussion

In the present investigation, a heterozygous *PRRX1* variation, NM_022716.4:c.373G>T;p.(Glu125^*^), was found to be in co-segregation with AF and PDA in a family. The truncating variation was neither detected in 612 referential chromosomes nor retrieved in the Single Nucleotide Polymorphism database, the Single Nucleotide Polymorphism database, the Human Gene Mutation Database, the 1000 Genomes Project database or the UK Biobank database. Functional research unveiled that Glu125^*^-mutant *PRRX1* lost transcriptional activation on the promoters of *SHOX2* and *ISL1*, two genes where variations have been discovered to result in AF and CHD (Blaschke *et al.*, 2007; Hoffmann *et al.*, 2016, 2019; Li *et al.*, 2018; Ma *et al.*, 2019; Wang *et al.*, 2019; Wu *et al.*, 2020). These findings indicate that genetically defective *PRRX1* contributes to AF and PDA in this family.

In humans, *PRRX1* is localized to chromosome 1q24.2 and encodes paired related homeobox 1, as a member of the paired homeobox-containing family of transcription factors (Grueneberg *et al.*, 1992). The *PRRX1* protein is highly expressed in the cardiovascular system throughout embryonic development, predominantly in the mesenchymal tissues, including the heart, endothoracic great arteries and pulmonary veins (Leussink *et al.*, 1995; Bergwerff *et al.*, 1998; Libório *et al.*, 2011), a common source of ectopic beats triggering AF in the majority of patients (Haïssaguerre *et al.*, 1998). It has been validated that *PRRX1* regulates the epithelial to mesenchymal transition, a hallmark of human cardiovascular morphogenesis (Ocaña *et al.*, 2012). Notably, in *PRRX1*-knockout mice, cardiovascular developmental malformations occurred, encompassing awkward curvature and abnormal positioning of the aortic arch, an aberrant retro-esophageal right subclavian artery as well as a misdirected and elongated ductus arteriosus, highlighting the crucial role of *PRRX1* in the proper development of vessels and perivascular matrices (Bergwerff *et al.*, 2000). Moreover, a recent study has demonstrated that *PRRX1* physically binds to the promoters of *SHOX2* and *ISL1* and transcriptionally activates the expression of *SHOX2* and *ISL1* (Guo *et al.*, 2021), two key downstream target genes responsible for the normal development of the heart, especially for its pacing and conducting system (Blaschke *et al.*, 2007; Liang *et al.*, 2015; Vedantham *et al.*, 2015; Galang *et al.*, 2020) and variations in both *SHOX2* and *ISL1* have been causally linked to AF and CHD (Blaschke *et al.*, 2007; Hoffmann *et al.*, 2016, 2019; Li *et al.*, 2018; Ma *et al.*, 2019; Wang *et al.*, 2019; Wu *et al.*, 2020). In the present research, a new *PRRX1* loss-of-function variation was discovered to lead to AF and PDA. Collectively, these observational results support that *PRRX1* haploinsufficiency is involved in the molecular pathogenesis of AF and CHD in some cases.

Recently, multiple genome-wide association studies and a meta-analysis consistently revealed that a common single nucleotide polymorphism (rs3903239) about 63 kb upstream of the *PRRX1* gene, a top genetic variation at the locus of AF on chromosome 1q24, was associated with significantly increased risk of AF in both Europeans and Asians (Tucker *et al.*, 2017; Wu *et al.*, 2021). Functional analyses unveiled that this variant diminished the transcriptional activity of the promoter of *PRRX1*, resulting in reduced expression of *PRRX1* in human left atrial tissue (Tucker *et al.*, 2017). Moreover, loss of *PRRX1* was shown to shorten the action potential duration as well as effective refractory period in human atrial cardiomyocytes and zebrafish embryonic myocardium, forming a substrate vulnerable to AF (Tucker *et al.*, 2017), which was further substantiated in a mouse model with deletion of the noncoding AF-associated genomic region (Bosada *et al.*, 2021). Additionally, two loss-of-function variations in *PRRX1* have been uncovered to cause familial AF (Guo *et al.*, 2021). In this research, a new *PRRX1* loss-of-function variation was identified to give rise to AF and PDA, therefore expanding the phenotypic spectrum linked to *PRRX1* and supporting *PRRX1* as a causative gene for AF and CHD. Notably, heterozygous loss-of-function variations in *PRRX1* have already been described in patients with agnathia-otocephaly complex, a rare condition characterized by mandibular hypoplasia or agnathia, ear anomalies (melotia/synotia) and microstomia with aglossia (Dubucs *et al.*, 2021). It is interesting that the same kind of variation has been associated with a quite different phenotype (AF) in the present study, which may be explained in part by the distinct genetic backgrounds.

## Conclusions

This study firstly associates *PRRX1* loss-of-function variation with AF and PDA in humans, which suggests that AF and PDA may share a common basis of anomalous cardiovascular development in a subset of cases, implying potential implications for early precise prophylaxis and improved prognostic risk stratification of patients affected with AF and PDA.
